# β-Glucan training induces stimulus-dependent immune and metabolic modulation in head kidney leukocytes that persists for several days in Atlantic salmon (*Salmo salar* L.)

**DOI:** 10.3389/fimmu.2026.1815436

**Published:** 2026-05-07

**Authors:** Thinh Hoang Nhan, Lluis Tort, Carlo C. Lazado

**Affiliations:** 1 Nofima, The Norwegian Institute of Food, Fisheries and Aquaculture Research, Ås, Norway; 2 Department of Cell Biology, Physiology and Immunology, Universitat Autònoma de Barcelona, Barcelona, Spain; 3 Faculty of Bioscience Engineering, Ghent University, Gent, Belgium

**Keywords:** aquaculture, immunological memory, immunostimulant, phagocytosis, trained immunity

## Abstract

The innate immune system of fish has conventionally been considered incapable of immunological memory, but is now being recognised as exhibiting memory-like features known as trained immunity. This study investigated the induction of trained immunity in Atlantic salmon (*Salmo salar* L.) by training head kidney-derived leukocytes using β-glucan, followed by a resting phase and secondary stimulation with β-glucan (homologous) or lipopolysaccharide (LPS, heterologous). The cellular responses, metabolite production, and gene expression related to innate immunity, metabolism, and epigenetic markers were assessed. The effects of initial β-glucan training persisted after a 5-day resting period, during which upregulation occurred in the expression of key innate immune and metabolic genes. Upon secondary stimulation, leukocytes exhibited stimulus-dependent transcriptional responses with increased expression of several pro-inflammatory and metabolic genes, particularly in the heterologous LPS-exposed group. Attenuation of specific inflammatory cytokine responses occurred in trained cells upon LPS stimulation, but metabolic gene expression patterns indicated regulation toward enhancing glycolytic activity and mitochondrial oxidative metabolism. Trained cells also displayed significantly increased phagocytic activity, especially after heterologous exposure. Only minor or moderate changes occurred in other cellular outputs (reactive oxygen species, nitric oxide, lactate, and fumarate). Epigenetic markers showed limited expression changes. The experimental evidence indicates a phenotype similar to trained immunity in salmon leukocytes, characterised by transcriptional and functional alterations following β-glucan training; however, responses vary upon secondary exposure to a heterologous stimulus. This study provides new insight into trained immunity in Atlantic salmon by demonstrating the transcriptional and cellular response of leukocytes to develop stimulus-dependent immune and metabolic regulations.

## 1 Introduction

Conventionally, immune memory is regarded as a distinctive feature of the adaptive immune system. However, recent studies have demonstrated that innate immune cells can also develop memory-like properties through trained immunity (1–5). Unlike adaptive memory, trained immunity is mediated by epigenetic and metabolic reprogramming (3, 4). This discovery has redefined the classical view of innate immunity as purely nonspecific and highlights its capacity for functional adaptation and long-term modulation of host defence.

Trained immunity was first described in mammals. In humans, Bacillus Calmette–Guérin vaccination has been shown to induce trained immunity in innate immune cells by activating the Akt–mTOR–HIF-1α pathway. This process promotes glycolysis and histone modifications that increase cytokine production and are correlated with reduced viral viremia (6, 7). Moreover, Bacillus Calmette–Guérin administration imprints long-lasting transcriptional and epigenetic changes in hematopoietic progenitors that functionally enhance the innate immune profile (8). In mice, exposure to *Candida albicans* or zymosan A enhances immune protection to secondary bacterial infections through IL-1β-dependent activation (2), which even persists across generations through germline epigenetic changes (9). β-glucan priming *in vitro* also enhances murine monocyte survival and cytokine responses, but the effect is transient and short-lived *in vivo* (10).

Growing evidence supports the existence of trained immunity in teleost fish, indicating that key features of this mechanism are conserved across early vertebrates, including fish (11, 12). Larvae of zebrafish (*Danio rerio*) that have been trained with sub-lethal doses of live or heat-killed *Salmonella typhimurium* show protection against lethal doses of the same pathogens and unrelated ones in a secondary challenge (13). In addition, β-glucan priming in zebrafish and embryonic-derived cell lines enhances neutrophil recruitment, viral resistance, and cytokine expression both *in vivo* and *in vitro* (13, 14). In macrophages of common carp (*Cyprinus carpio*), β-glucan training boosts phagocytic activity, inflammatory responses, and the expression profiles of metabolic and epigenetic markers and leads to metabolic shifts toward glycolysis, as indicated by an accumulation of metabolites (e.g., lactate and fumarate) (12, 15).

*In vivo* studies on turbot (*Scophthalmus maximus*) and channel catfish (*Ictalurus punctatus*) have shown that β-glucan exposure induces metabolic changes and epigenetic reprogramming associated with trained immunity, such as enhanced survival, phagocytic activity, and neutrophil responses, as well as specific histone modifications in head kidney (HK) leukocytes, including H3K4me1, H3K4me3, H3K27ac, and H3K27me3 (16–18). In salmonid models *in vivo*, trained immunity-like responses have been demonstrated in rainbow trout (*Oncorhynchus mykiss*), where heat-killed *Mycobacterium marinum* or muramyl dipeptide induced long-term, non-specific protection against bacterial infection. These effects were associated with improved proinflammatory cytokine expression and epigenetic activation marks (H3K4me3, H3K27ac) in HK leukocytes (19, 20).

Immunostimulants such as β-glucans are widely used in aquaculture to enhance innate immunity, and their properties and long-lasting effects make them ideal candidates for inducing trained immunity (11, 21, 22). β-glucan treatment *in vitro* maintains and improves Atlantic salmon HK macrophage respiratory burst activity up to 7 days, indicating a sustained priming effect (23). Soluble β-glucan activates HK leukocytes in salmon and induces strong inflammatory gene expression while reducing cell death (24). Understanding how β-glucans reprogram innate cells could support disease-control strategies, particularly in early life stages when adaptive responses are underdeveloped, and vaccination is not yet applicable. In Atlantic salmon, however, trained immunity and the utilisation of β-glucans to induce this mechanism have been largely unstudied.

This study investigates whether β-glucan induces trained immunity features in Atlantic salmon HK leukocytes. Establishing whether this phenomenon occurs in Atlantic salmon is important for comparative immunology across teleosts and other species. The focus is on immune functions, metabolic activity, and gene expression related to innate immunity, metabolism, and epigenetics. We hypothesised that β-Glucan training causes a persistent priming of the leukocytes (for several days) that is specific to both immunity and metabolism, such that trained cells show improved functional and metabolic responses compared with untrained controls.

## 2 Materials and methods

### 2.1 Ethics statement

All fish-handling procedures complied with the European Union Directive 2010/63/EU and the Norwegian Animal Welfare Act of 2009. The experimental fish were outsourced from the fish production stock of the Centre for Fish Trials at the Norwegian University of Life Sciences (NMBU; Ås, Norway). Fish at the station undergo routine screening for key pathogens; individuals used in the trial tested negative for all known pathogens. The fish were not subjected to any pain or distress and were euthanised solely for cell isolation. Approval from the Norwegian Food Safety Authority was not required for this purpose. The personnel who sampled the tissue have a FELASA C certificate.

### 2.2 Isolation of head kidney leukocytes from Atlantic salmon

**Figure 1:**
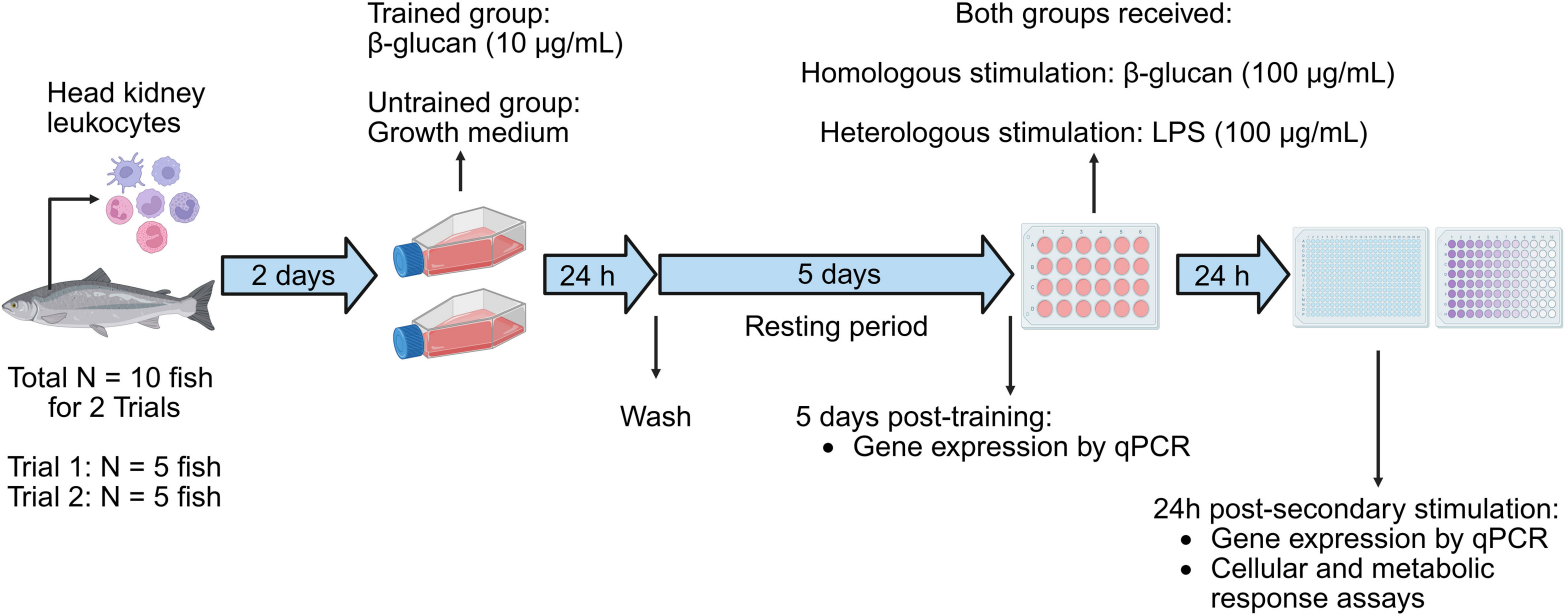
Diagram of the experimental design. HK leukocytes were isolated from Atlantic salmon (N = 5 per trial, total N = 10 for 2 trials) and cultured for 2 days before primary stimulation with β-glucan for 24 h. After a 5-day resting phase, cells were subjected to secondary stimulation with either β-glucan (homologous) or LPS (heterologous) for 24 h. Cellular responses and gene expression analyses were then performed. The diagram was created using https://BioRender.com/3p9kfsk.

A schematic of the experimental design is described in **Figure 1**. Two trials were conducted to isolate HK leukocytes. Trial 2 was conducted three weeks after Trial 1, using the same fish batch from NMBU. In Trial 1, HK leukocytes were used to evaluate nitric oxide (NO) production, phagocytic activity, reactive oxygen species (ROS) generation, intracellular lactate production, and to perform real-time quantitative polymerase chain reaction (RT-qPCR) for gene expression analysis. In Trial 2, HK leukocytes were used to assess fumarate concentration and to conduct RT-qPCR for confirmation of gene expression profiles. For RT-qPCR, data from both trials were pooled to increase statistical power, as all fish originated from the same batch and were maintained under identical conditions and subsequently subjected to the same cell isolation procedure.

Unvaccinated freshwater Atlantic salmon (ca 250 g) were euthanised using an overdose of Aqui-S anaesthetic. HK leukocytes were isolated and cultured in Leibovitz’s L-15 Medium + GlutaMAX™ (L-15 medium; Thermo Fisher Scientific) according to a modification of a published protocol (25). Briefly, the HK leukocytes isolation from fish (*N=*5 per trial) was collected and suspended in L-15 medium with 1% Antibiotic-Antimycotic (Thermo Fisher), 2% foetal bovine serum (FBS; Thermo Fisher Scientific), and 0.4% heparin (20 U/mL) at 4°C. Tissues were gently macerated and passed through a 100-μm Falcon™ cell strainer (Corning). The cell suspension was layered on a 40:60% Percoll gradient and centrifuged at 500 × *g* for 30 min at 4 °C.

Leukocytes at the interphase were collected and washed twice with ice-cold L-15 medium containing 5% FBS. Cells were resuspended in growth medium (L-15 + 10% FBS, 1% Antibiotic-Antimycotic, 1% HEPES; Sigma-Aldrich), and viability was assessed using 0.4% Trypan Blue (Sigma-Aldrich) and a Countess Automated Cell Counter (Thermo Fisher Scientific). The cells density was adjusted to 1 × 10^6^ cells/50 mL, seeded separately into 25-cm² flasks for trained and untrained groups to use for downstream processing, and incubated at 10 ± 1 °C under constant darkness for 48 h to allow recovery and attachment.

### 2.3 β-glucan training and secondary stimulation of HK leukocytes

After 48 h, HK leukocytes were washed with Dulbecco’s phosphate-buffered saline (DPBS; Thermo Fisher Scientific) to remove debris. Cells in the trained group were then treated with 10 mL of growth medium containing 10 µg/mL β-glucan (from *Saccharomyces cerevisiae*; Merck KGaA), a concentration previously shown to induce immune responses in fish cells without overactivation (12, 14). An untrained control group was treated using growth medium without β-glucan. The cells were incubated for 24 h to induce training, then washed with 10 mL of DPBS to remove β-glucan residues and dead cells. The untrained group was handled similarly. HK leukocytes were rested for 5 days at 10 ± 1 °C under constant darkness. This resting period was selected based on the range previously reported in teleost trained immunity studies (11).

After the resting phase, adherent cells were detached using 0.25% trypsin-EDTA (Sigma-Aldrich) then neutralised with growth medium. Cells were gently scraped, centrifuged (500 × *g*, 5 min), and resuspended in 10 mL of growth medium. A 300-µL suspension was collected from each flask for RT-qPCR analysis at 5 days post-training. Cell viability was checked before seeding onto new plates.

For the secondary stimulation phase, phagocytosis, ROS generation, intracellular lactate quantification, and fumarate production were performed using 96-well plates. In these assays, 100 µL of HK leukocyte suspension was dispensed into each well, and all treatments were run in duplicate. Each plate included four treatment groups: a no-treatment control (150 µL of growth medium, no cells), a media control (100 µL of cells + 50 µL of growth medium), a homologous stimulation group (100 µL of cells + 50 µL of growth medium with 100 µg/mL β-glucan), and a heterologous stimulation group (100 µL of cells + 50 µL of growth medium with 100 µg/mL lipopolysaccharide (LPS from *Escherichia coli*; Sigma-Aldrich)). The plates were incubated for 24 hours at 10 ± 1 °C before downstream assays.

The β-glucan and LPS concentrations used were based on previous studies (12, 26). A higher dose (100 µg/mL) of either β-glucan or LPS was used for secondary stimulation to assess stimulus-specific responses. LPS was chosen to evaluate cross-protection as it activates pattern-recognition pathways distinct from the C-type lectin receptor pathways activated by β-glucan (21). Moreover, several studies have demonstrated that LPS can induce an immune response in Atlantic salmon via Toll-like receptor (TLR)-independent pathways (26–28).

For gene-expression analysis and NO production assays, 24-well plates were used to accommodate larger cell volumes, with all treatments also performed in duplicate. In these experiments, 300 µL of leukocyte suspension was added to each well. One plate served as a control and contained only cells plus 200 µL of growth medium per well. A second plate was prepared for stimulation conditions, in which each well contained cell suspension and 200 µL of growth medium containing either β-glucan or LPS with a stimulant concentration of 100 µg/mL. These plates were also incubated for 24 hours at 10 ± 1 °C before sample collection.

### 2.4 Cellular responses following β-glucan training and secondary stimulations

#### 2.4.1 Phagocytosis

Phagocytic activity was assessed using the Vybrant™ Phagocytosis Assay Kit (Thermo Fisher Scientific; Cat: V6694). After secondary stimulation, HK leukocyte medium (see section 2.3) was removed and replaced with 100 µL of fluorescein-labelled *E. coli* BioParticle suspension in each well. Cells were incubated at 10 ± 1 °C for 2 hours to induce phagocytosis. Following incubation, the wells were washed to remove the BioParticle suspension, and 100 µL of diluted trypan blue solution was added to each well for 1 minute, after which it was removed. Fluorescence intensity was measured using a microplate reader at 480 nm excitation and 520 nm emission, and phagocytosis was calculated according to the kit’s instructions.

#### 2.4.2 Reactive oxygen species generation

Intracellular ROS levels were measured using a Fluorometric Intracellular ROS Assay Kit (Sigma-Aldrich; Cat: MAK143) with some modifications. In each well of the HK leukocyte plates (see section 2.3), 100 µL of Master Reaction Mix containing ROS Detection Reagent was added, followed by incubation for 2 hours at 12 °C. After incubation, 20 µL of treatments (LPS, β-glucan, or growth medium) was added to each well, and the cells were incubated again at 10 °C for 24 h. The fluorescence intensity was measured at 490 nm excitation and 525 nm emission.

#### 2.4.3 Nitric oxide production

Total NO was measured via its stable end-products, nitrite (NO_2_^-^) and nitrate (NO_3_^-^), using a Nitric Oxide Assay Kit (Sigma-Aldrich; Cat: MAK454) with minor modifications. After secondary stimulation (see section 2.3), HK leukocytes were scraped, centrifuged (500 *× g*, 10 min, 4 °C), resuspended in 300 µL of DPBS, vortexed, and sonicated (3 sec). Samples were deproteinated with 8 µL each of NaOH and ZnSO_4_, vortexed, and centrifuged (14,000 *× g*, 10 min). 100 µL of supernatant was mixed with 200 µL of Working Reagent (including Griess and catalyst reagents) and incubated at 37 °C for 60 min. 250 µL of the reaction mixture was then transferred to a 96-well plate, and the absorbance was measured at 540 nm. The NO level was calculated according to the kit’s instructions.

#### 2.4.4 Intracellular lactate quantification

Intracellular lactate was quantified using the Lactate-Glo™ Assay (Promega; Cat: J5021) according to the manufacturer’s protocol for cell lysates. After secondary stimulation (see section 2.3), HK leukocytes were washed twice with 200 µL of DPBS, resuspended in 25 µL of DPBS, and treated with 12.5 µL of inactivation solution (0.6 N HCl). The plates were shaken for 5 min, followed by the addition of 12.5 µL of neutralisation solution (1 M Tris base, pH ~10.7) and brief shaking (30–60 sec). Then, 50 µL of lactate detection reagent was added, and the plate was shaken again and incubated for 60 min at room temperature. Luminescence was recorded using a plate-reading luminometer.

#### 2.4.5 Fumarate production

The fumarate concentration was detected based on enzymatic reactions using an EnzyChrom™ Fumarate Assay Kit (BioAssay Systems; Cat: EFUM-100). HK leukocytes after secondary stimulation (see section 2.3) were rinsed with DPBS and homogenised in 200 µL of assay buffer and centrifuged at 10,000 *× g* for 15 min at 4 °C. The supernatant was collected, and 20 µL were transferred to a separate 96-well plate. 80 µL of working reagent (containing Assay Buffer, NAD/MTT Solution, Catalyst Enzymes, and FMR Enzyme) was added to the plate and incubated for 30 minutes at room temperature. The absorbance was then read at 565 nm (OD_565nm_), and the fumarate concentration was calculated according to fumarate standards.

### 2.5 RNA isolation, cDNA synthesis, and RT-qPCR

Total RNA was extracted using a Quick-RNA Microprep kit (ZymoResearch; Cat: R1051). HK leukocytes were washed with DPBS, 350 µL of lysis buffer was added, and the cells were scraped and used for RNA isolation by following the manufacturer’s protocol. The concentration and purity of RNA were checked using a NanoDrop 8000 Spectrophotometer (Thermo Fisher Scientific). cDNA was synthesised from 5 ng/μL of normalised RNA using a High-Capacity cDNA Reverse Transcription Kit (Thermo Fisher Scientific; Cat: 4374967) in a 20-μL reaction volume. Reverse transcription PCR was performed using a Veriti™ 96-Well Thermal Cycler (Applied Biosystems) with the following thermocycling procedure: 25 °C for 10 min, 37 °C for 120 min, 85 °C for 5 min, and then held at 4 °C.

RT-qPCR was performed using the QuantStudio™ 5 Real-Time PCR System. Each PCR reaction consisted of 4 μL of 10× diluted cDNA, 5 μL of SYBR Green Master Mix, and 0.5 μL of 10 μM forward and reverse primers for the reference/target gene (**Supplementary File 1**). The thermocycling process was as follows: an initial step at 50 °C for 2 min, followed by a denaturation phase at 95 °C for 2 min, then 40 amplification cycles comprising 1 sec at 95 °C and 30 sec at 60 °C, and finishing with a melt-curve stage with steps at 95 °C for 15 sec, 60 °C for 1 min, and 95 °C for 15 sec.

Primer efficiencies were assessed using a five-point standard curve generated from a two-fold serial dilution of pooled cDNA (1 µL from each sample). All reactions were run in duplicate with minus reverse transcriptase and no-template controls. The relative gene expression of the target gene was calculated using the 2^-ΔΔCt^ method, normalised to the geometric mean of three housekeeping genes: *18S ribosomal RNA* [*18s*], *elongation factor 1-α* [*elf1a*], and *ubiquitin* [*ubi*].

### 2.6 Statistical analysis

Statistical analyses were conducted using GraphPad Prism for Windows^®^ (version 9.0). An unpaired *t*-test was used to compare gene expression between trained and untrained leukocyte groups at 5 days post-training. For comparisons of secondary stimulations, two-way analysis of variance (ANOVA) was performed after checking for normality and homogeneity of variances to analyse differences in cellular responses, metabolic activity, and gene expression among treatment groups. If the assumptions of normality were not met after data transformation, the Kruskal–Wallis test was used, followed by Dunn’s multiple-comparisons test for pairwise group comparisons. In cases of unequal variances, Welch’s one-way ANOVA was applied, and Dunnett’s test was used for *post hoc* analysis. Statistical significance was set at *P* < 0.05.

## 3 Results

### 3.1 Gene-expression profiles of HK leukocytes 5 days post-training

**Figure 2:**
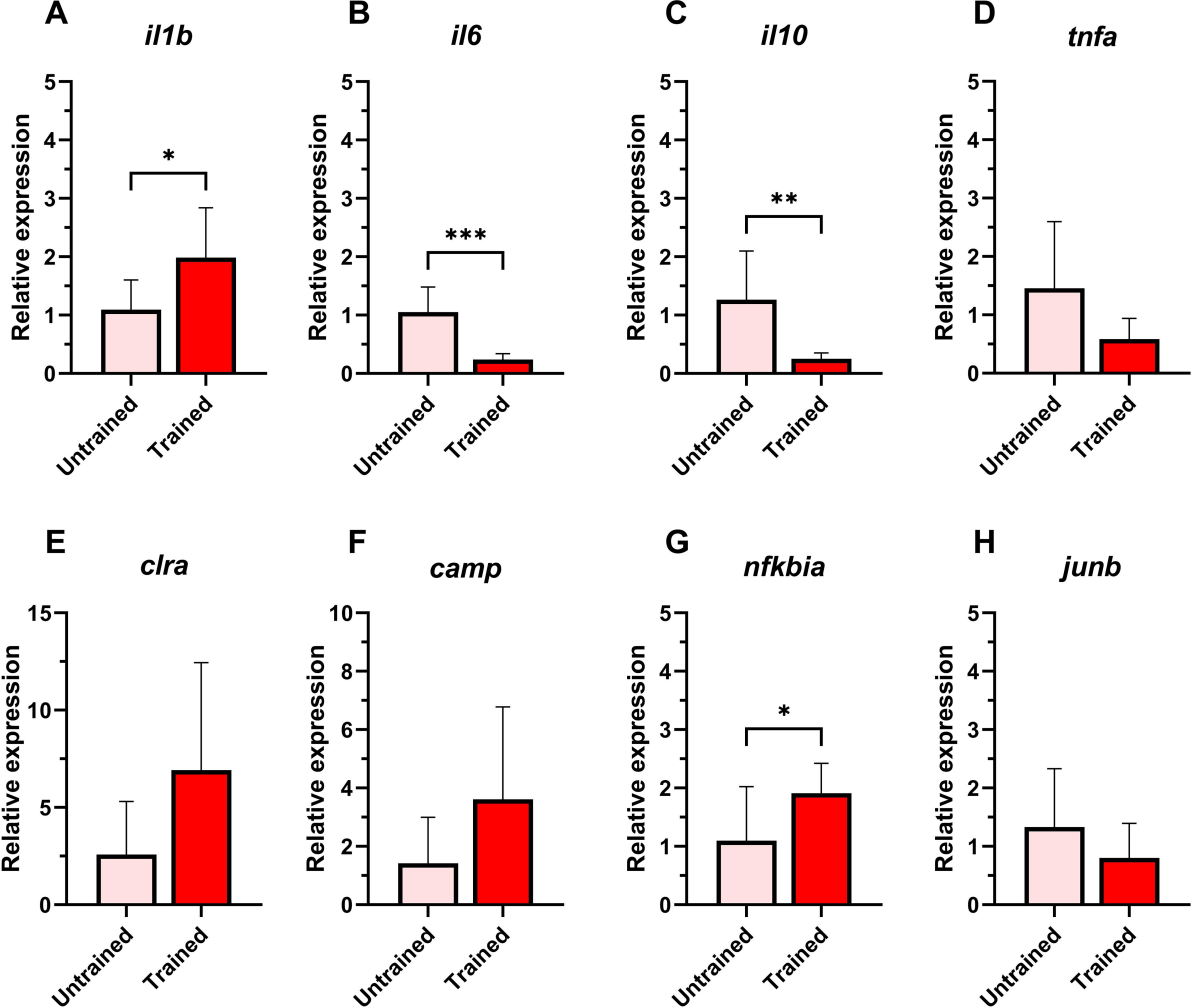
Relative expression of innate immune genes in Atlantic salmon HK leukocytes at 5 days post-training. **(A)** *il1b*, **(B)** *il6*, **(C)** *il10*, **(D)** *tnfa*, **(E)** *clra*, **(F)** *camp*, **(G)** *nfkbia*, **(H)** *junb*. Values are presented as mean + standard deviation of relative gene expression values from HK leukocytes isolated from 10 individual fish (N = 10) from Trial 1 and 2, where 5 fish were used per trial. A significant difference between the two groups is indicated with an asterisk: *p < 0.05; **p < 0.01; ***p < 0.001.

At 5 days following β-glucan training, trained leukocytes showed a significant upregulation of *il1b* and *nfkbia* compared to untrained cells (**Figures 2A, G**). In contrast, trained leukocytes exhibited significantly lower expression of *il6* and *il10* relative to untrained cells (**Figures 2B, C**). No significant differences were detected in *tnfa* and *junb* expression between groups (**Figures 2D, H**). *clra* and *camp* showed higher expression in trained cells, but these changes were not significant compared to the untrained group (**Figures 2E, F**).

**Figure 3:**
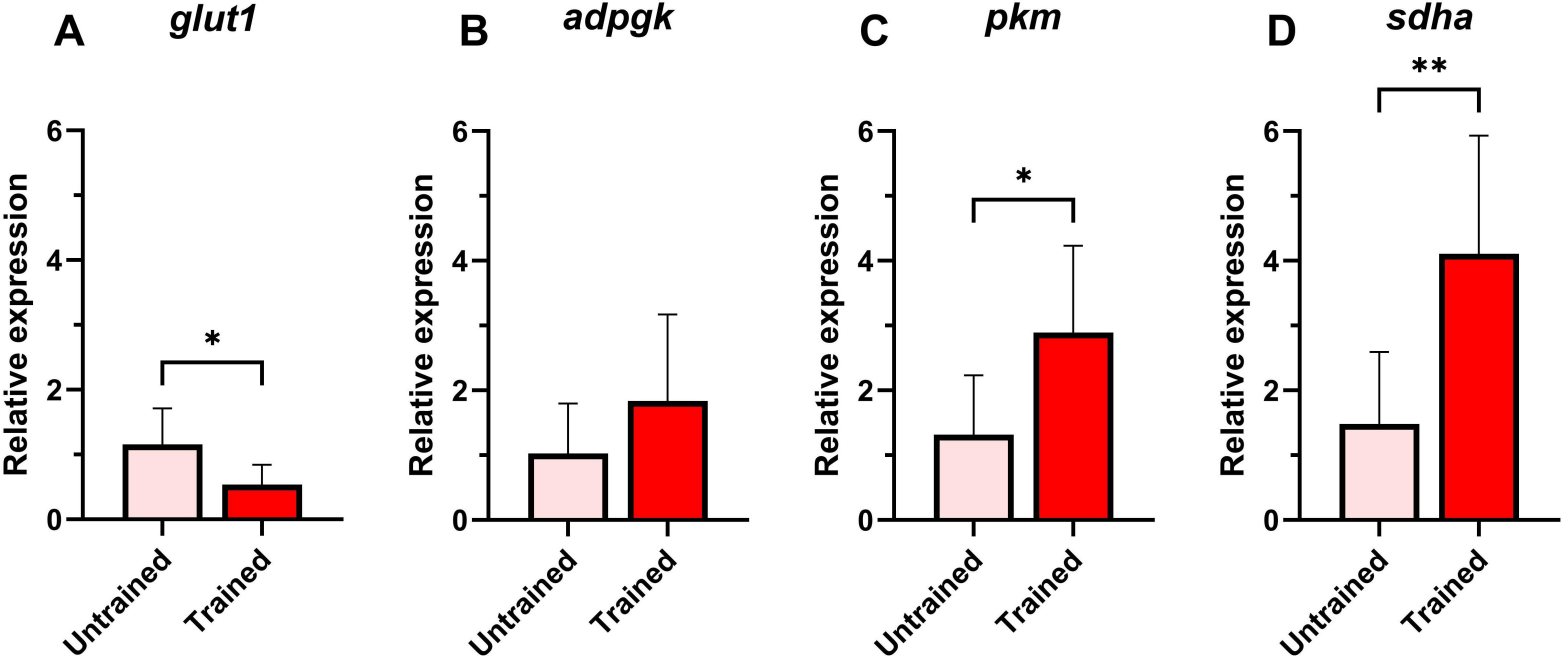
Relative expression of metabolism-related genes in Atlantic salmon HK leukocytes at 5 days post-training. **(A)** *glut1*, **(B)** *adpgk*, **(C)** *pkm*, **(D)** *sdha*. Values are presented as mean + standard deviation of relative gene expression values from HK leukocytes isolated from 10 individual fish (N = 10) from Trial 1 and 2, where 5 fish were used per trial. A significant difference between the two groups is indicated with asterisks: *p < 0.05; **p < 0.01.

Most metabolism-related genes showed increased expression in the trained group compared to the untrained one. The expressions of *sdha* and *pkm* were notably upregulated in trained leukocytes (**Figures 3C, D**), while *adpgk* had no significantly different expression compared to the untrained group (**Figure 3B**). However, *glut1* expression was significantly lower in trained cells compared to the untrained (**Figure 3A**).

**Figure 4:**
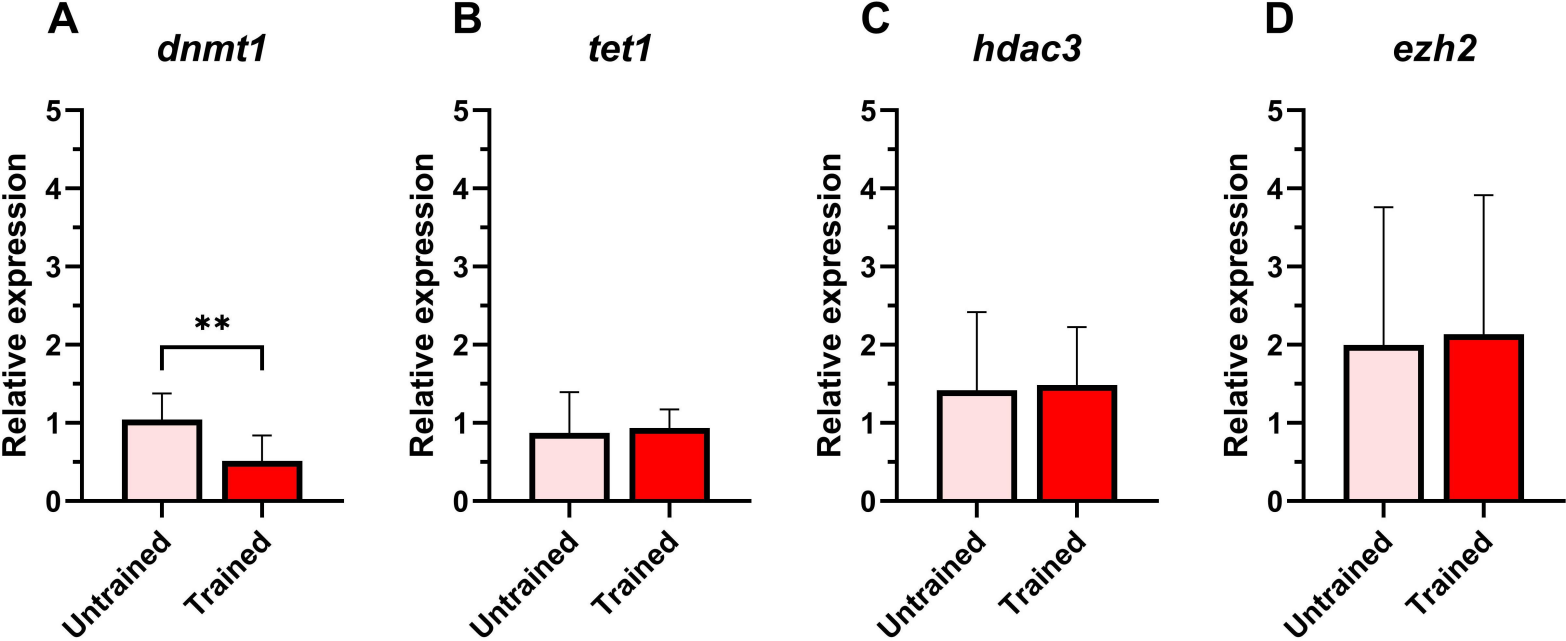
Relative expression of epigenetic-related genes in Atlantic salmon HK leukocytes at 5 days post-training. **(A)** *dnmt1*, **(B)** *tet1*, **(C)** *hdac3*, **(D)** *ezh2*. Values are presented as mean + standard deviation of relative gene expression values from HK leukocytes isolated from 10 individual fish (N = 10) from Trial 1 and 2, where 5 fish were used per trial. A significant difference between the two groups is indicated with asterisks: **p < 0.01.

Modest trends were observed in four epigenetic marker genes. The expression of *dnmt1* was significantly downregulated in trained leukocytes compared to the untrained group (**Figure 4A**). The expression levels of *tet1, hdac3*, and *ezh2* showed no significant differences between the two groups (**Figures 4B–D**).

### 3.2 Gene-expression profiles of HK leukocytes 24 hours after secondary stimulations

**Figure 5:**
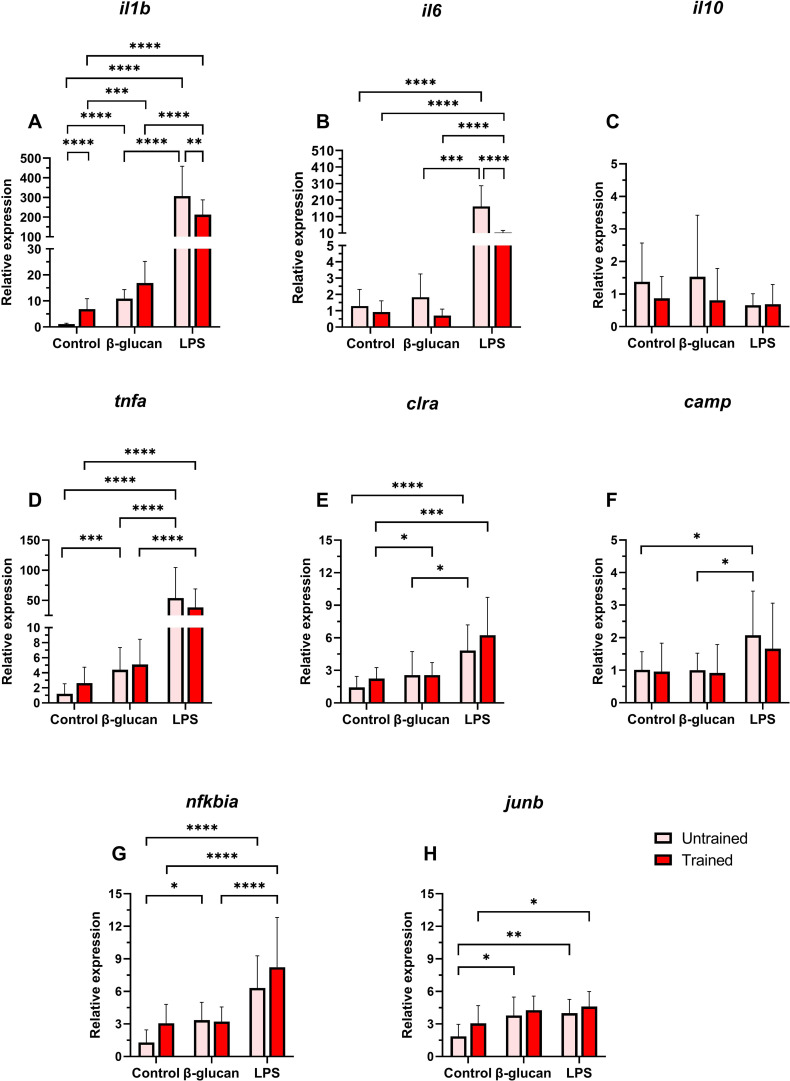
Relative expression of innate immune genes in Atlantic salmon HK leukocytes at 24 hours after secondary stimulations. **(A)** *il1b*, **(B)** *il6*, **(C)** *il10*, **(D)** *tnfa*, **(E)** *clra*, **(F)** *camp*, **(G)** *nfkbia*, **(H)** *junb*. Values are presented as mean + standard deviation of relative gene expression values from HK leukocytes isolated from 10 individual fish (N = 10) from Trial 1 and 2, where 5 fish were used per trial. A significant difference between the two groups is indicated with asterisks: *p < 0.05; **p < 0.01; ***p < 0.001; ****p < 0.0001.

At 24 h after secondary stimulation, immune gene expression showed clear stimulus-dependent responses. LPS significantly upregulated *il1b*, *il6*, and *tnfa* in both trained and untrained cells compared to β-glucan and control treatments (**Figures 5A, B, D**). However, trained leukocytes had lower *il1b* and *il6* expression under LPS stimulation than untrained cells (**Figures 5A, B**). In the control group and with β-glucan stimulation, *il1b* expression was higher in trained cells (significant in control), while *il6* was slightly lower, although not significantly. *Il10* expression was consistently lower in trained cells across all treatments, although not significantly (**Figure 5C**).

Other immune genes also demonstrated stimulus-dependent transcriptional patterns with no significant difference between the trained and untrained groups. *Clra* and *nfkbia* had significantly elevated expression under LPS stimulation compared to the control and β-glucan treatments in both groups (**Figures 5E, G**). *Camp* expression was similar in both train and untrained groups in all treatments except for a significant upregulation in untrained cells under LPS stimulation (**Figure 5F**). *Junb* expression showed a modest increasing trend among stimulations, but no difference was observed between trained and untrained cells (**Figure 5H**).

**Figure 6:**
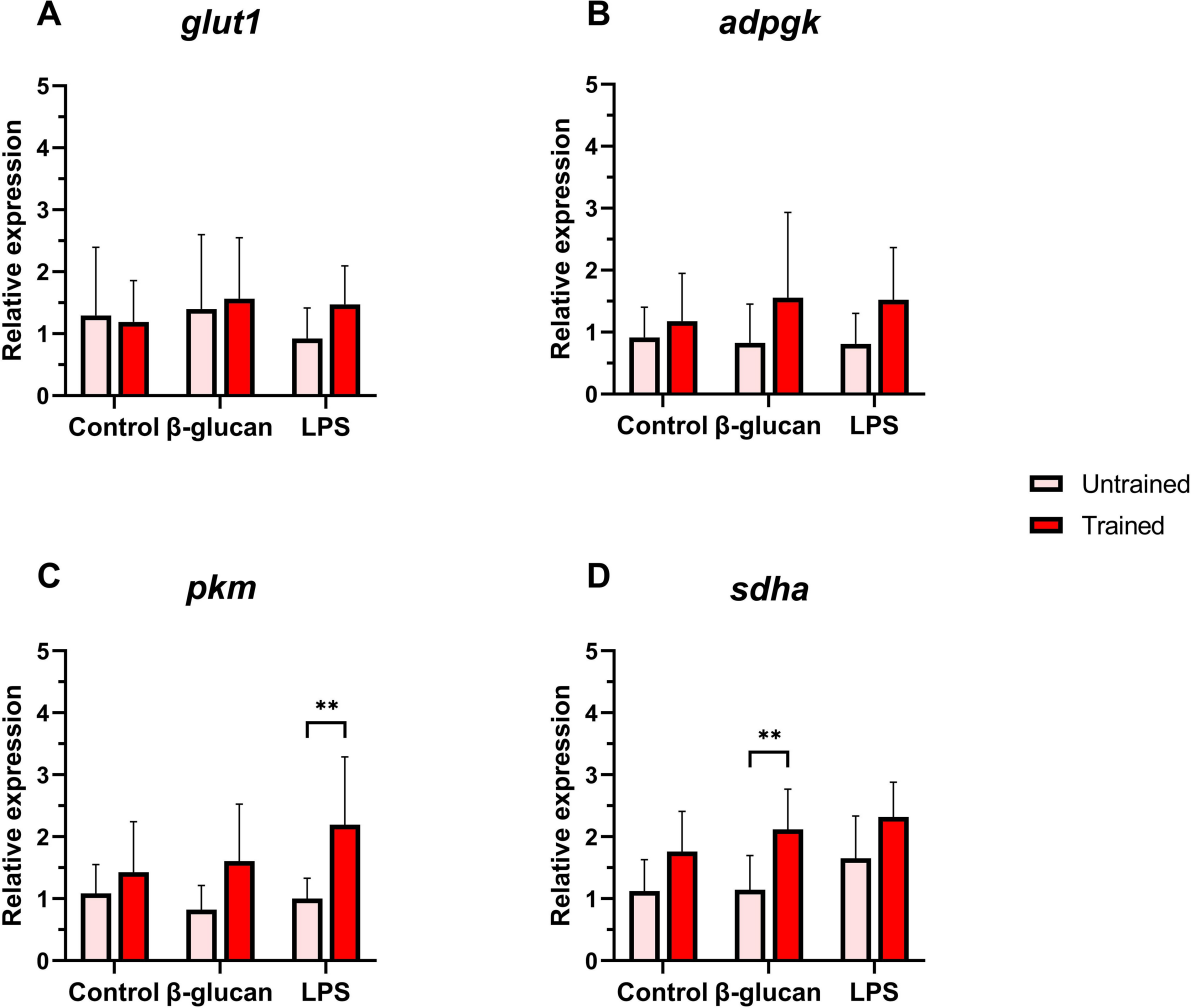
Relative expression of metabolism-related genes in Atlantic salmon HK leukocytes at 24 hours after secondary stimulations. **(A)** *glut1*, **(B)** *adpgk*, **(C)** *pkm*, **(D)** *sdha*. Values are presented as mean + standard deviation of relative gene expression values from HK leukocytes isolated from 10 individual fish (N = 10) from Trial 1 and 2, where 5 fish were used per trial. A significant difference between the two groups is indicated with asterisks: *p < 0.05; **p < 0.01.

The expression of cell metabolism-related genes showed stimulus- and training-specific responses following secondary stimulation (**Figure 6**). *Glut1* expression remained comparable between trained and untrained leukocytes across all conditions (**Figure 6A**). In contrast, *adpgk* expression appeared slightly elevated in trained cells compared to the untrained group, although not significantly (**Figure 6B**). *Pkm* expression was significantly upregulated in trained leukocytes after LPS stimulation (**Figure 6C**), while *sdha* expression was significantly higher in trained leukocytes after β-glucan stimulation (**Figure 6D**).

**Figure 7:**
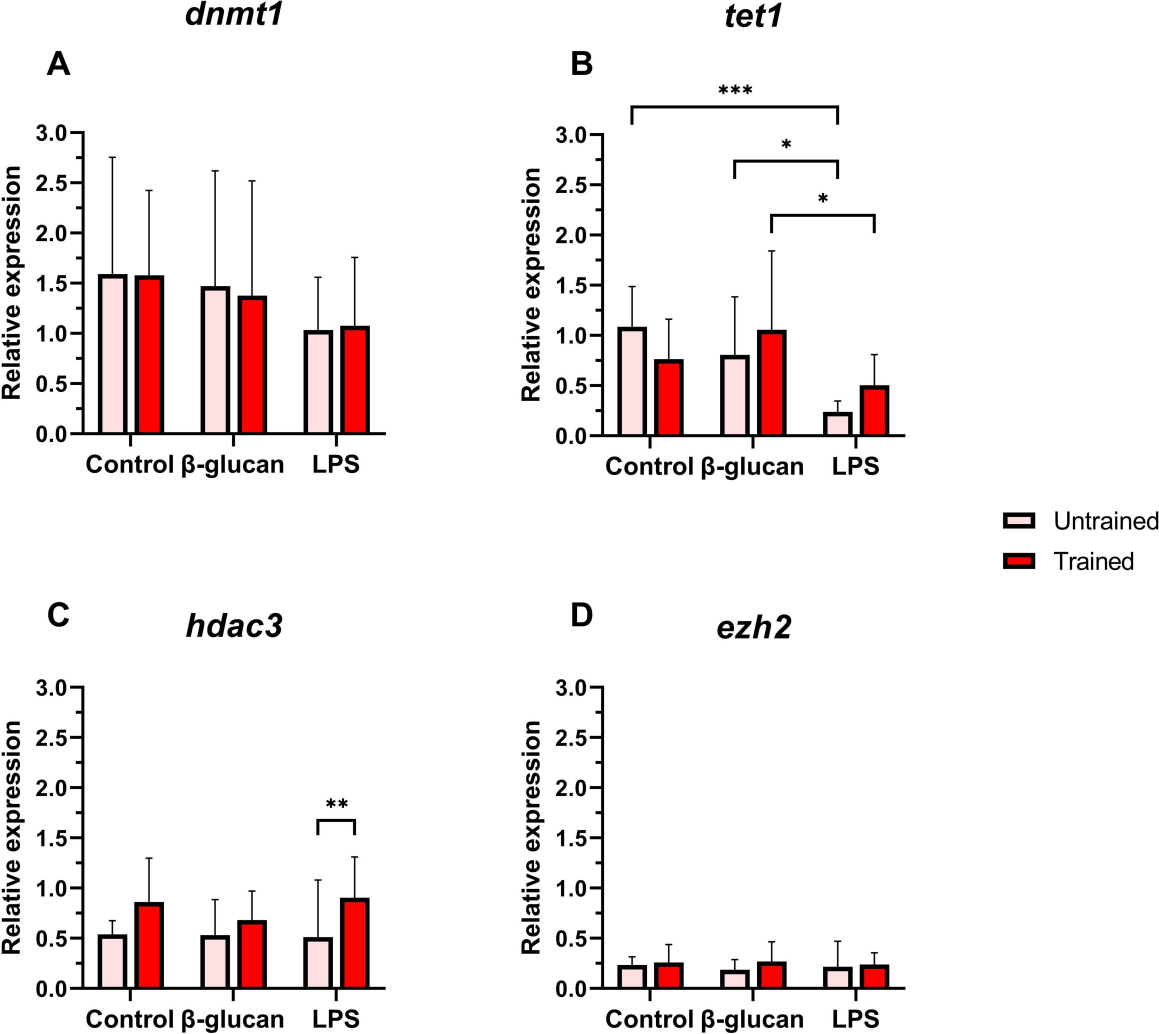
Relative expression of epigenetic-related genes in Atlantic salmon HK leukocytes at 24 hours after secondary stimulations. **(A)** *dnmt1*, **(B)** *tet1*, **(C)** *hdac3*, **(D)** *ezh2*. Values are presented as mean + standard deviation of relative gene expression values from HK leukocytes isolated from 10 individual fish (N = 10) from Trial 1 and 2, where 5 fish were used per trial. A significant difference between the two groups is indicated with asterisks: *p < 0.05; **p < 0.01; ***p < 0.001.

For epigenetic markers, gene-expression patterns showed limited changes after secondary stimulation (**Figure 7**). For *dnmt1* and *ezh2*, expression remained consistent across all conditions between trained and untrained leukocytes (**Figures 7A, D**). However, *dnmt1* showed a trend of lower expression in LPS-stimulated groups. LPS stimulation resulted in significantly lower *tet1* expression than in the β-glucan and control groups (**Figure 7B**). In addition, *hdac3* expression was higher in trained leukocytes across all treatments and with significance in the case of LPS (**Figure 7C**).

### 3.3 Cellular responses following β-glucan training and secondary stimulations

**Figure 8:**
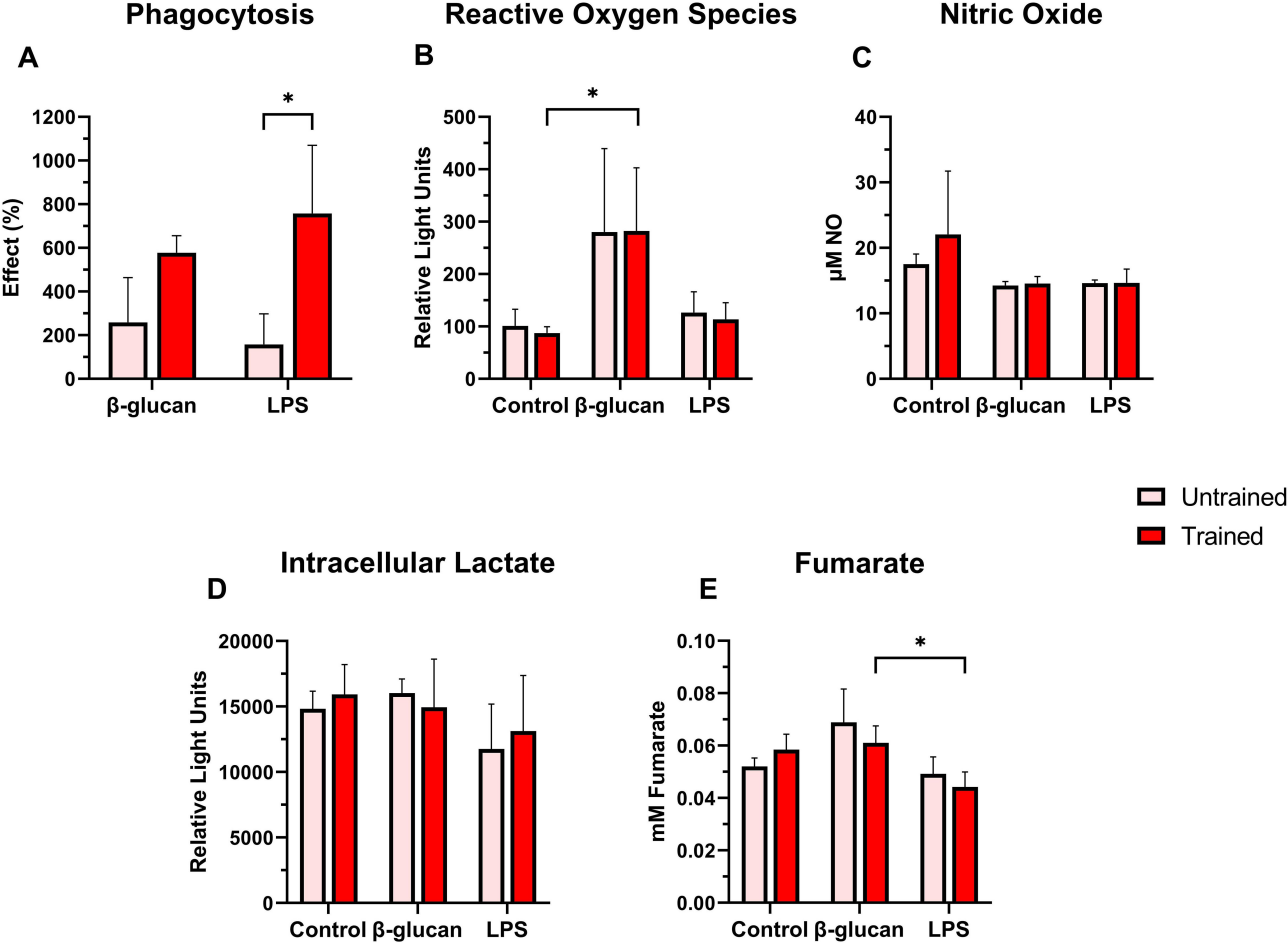
Cellular responses of HK leukocytes following β-glucan training and 24 hours after secondary stimulation. **(A)** Phagocytosis; **(B)** Reactive oxygen species generation; **(C)** Nitric oxide production; **(D)** Intracellular lactate quantification; **(E)** Fumarate production. Values are presented as mean + standard deviation of unit correspondence to each assay. HK leukocytes were isolated from 5 individual fish (N = 5). A significant difference between the two groups is indicated with an asterisk: *p < 0.05.

Following secondary stimulations, trained leukocytes showed higher phagocytic activity compared to untrained cells, especially significant under LPS stimulation, for which the average effect was approximately 4 times higher (**Figure 8A**). A similar trend was observed in response to β-glucan stimulation, although no statistically significant differences were detected. Regarding ROS production, no training effect was observed between trained and untrained leukocytes across all treatments (**Figure 8B**). β-glucan stimulation elicited a marked increase in ROS levels in both groups, particularly in the trained group, which showed a significant difference compared to the control condition. In contrast, LPS-stimulated leukocytes showed no significant difference from other treatments.

NO production did not significantly differ between trained and untrained leukocytes under any treatment (**Figure 8C**). NO production was comparable between groups following stimulation with either β-glucan or LPS. In the control group, NO levels were slightly higher in trained than in untrained leukocytes, although this difference was not statistically significant. Intracellular lactate levels remained unchanged between trained and untrained leukocytes across all treatments (**Figure 8D**). Following LPS stimulation, trained and untrained leukocytes showed no statistical significance, but they did exhibit moderately lower lactate levels than the other treatments.

Fumarate levels were also not affected by training, with trained and untrained leukocytes showing comparable production (**Figure 8E**). However, LPS-stimulated leukocytes exhibited lower fumarate levels than those in the β-glucan group, with this difference being especially significant in the trained groups. No significant difference was observed between the control and LPS treatments.

## 4 Discussion

Trained immunity is increasingly recognised as an important defence mechanism in fish. Unlike adaptive immunity, which depends on antigen-specific responses, it involves functional reprogramming of innate immune cells, resulting in a stronger response upon secondary challenge. Recent studies indicate that exposure to certain pathogens or immunostimulants can induce lasting changes in immune responsiveness, with potential benefits for disease resistance in aquaculture. In this study, we document this phenomenon in Atlantic salmon HK leukocytes using β-glucan as a training stimulus.

### 4.1 Trained leukocytes display distinct immune gene expression profiles after the resting phase and show stimulus-dependent transcriptional responses upon secondary stimulation

*Il1b* was upregulated in trained leukocytes at 5 days post-training and after control secondary stimulation, indicating a sustained transcriptional imprint and a persistently primed inflammatory state. A significant increase in *il1b* expression was also observed in trained leukocytes under homologous stimulation compared to the control, suggesting that the same stimulus can elicit an improved secondary immune response. This expression pattern is consistent with previous reports of β-glucan-induced trained immunity in teleosts, which showed a notable upregulation of *il1b.* Furthermore, it suggests that the IL1R signalling pathway plays a central role in mediating this process and may coordinate associated metabolic reprogramming (12, 14, 15, 17, 29).

The downregulation in *il6* and *tnfa* expression in trained leukocytes at 5 days post-training contrasted their established proinflammatory roles. Following secondary stimulation, *il6* remained suppressed, whereas *tnfa* exhibited modest upregulation under control and homologous conditions. In contrast, *il10* expression was consistently lower both post-training and after restimulation, suggesting limited immune regulation. Although *il6*, *il10*, and *tnfa* are typically upregulated in response to β-glucan stimulation in fish (11, 30), their regulation appears highly context-dependent and is influenced by timing, dosage, and paralogue-specific mechanisms (e.g., *il6a* and *il6b*) (12, 14, 31). Under heterologous LPS stimulation, trained leukocytes displayed attenuated cytokine expression compared with untrained controls, suggesting β-glucan-induced LPS cross-tolerance that mitigates excessive endotoxin-driven inflammation (32). Similar mechanisms have been reported in zebrafish (*D*. *rerio*) and common carp (*C*. *carpio*), in which cytokine expression varies with parameters of immunostimulant exposure (33, 34). Moreover, in the absence of a fully immune competence evaluation, it cannot be excluded that reduced cytokine expression also reflects aspects of immune suppression or transient exhaustion.

*Clra*, which encodes C-type Lectin Receptor (CLR) A, was upregulated in trained leukocytes at 5 days post-training and remained elevated after secondary stimulation. CLRs are key β-glucan receptors in fish, despite the absence of a Dectin-1 homologue (35). In Atlantic salmon, *clr* genes are upregulated in the distal intestine after oral β-glucan exposure, suggesting involvement in PAMP recognition and immune signalling (36). Upon LPS stimulation, *clra* expression also increased in both trained and untrained cells, which likely reflects broader bacterial sensing via CLRs (27). As most teleosts lack a TLR4 ortholog (including salmon), which is responsible for LPS recognition, their sensing of LPS differs from the pathway presented in mammals (26–28). However, alternative receptors such as NOD1 and scavenger receptor class B2a may contribute to LPS detection (37, 38). Another study on Atlantic salmon demonstrated increased expression of CLRs upon exposure to LPS or bacterial challenge, which supports the involvement of CLRs against unrelated pathogens (39).

*Nfkbia* is an inhibitor of NF-κB (IκBα) that is involved in controlling immune and stress responses and was upregulated in trained cells after β-glucan training and secondary stimulation, particularly with LPS. In salmonids, upregulation of *nfkbia, il1b*, and *il8* has been reported following treatment with zymosan (a glucan) in a cell line from chinook salmon (*Oncorhynchus tshawytscha*) (40). Another study in zebrafish (*D. rerio*) demonstrated that LPS exposure can suppress MyD88-dependent signalling, resulting in reduced NF-κB activation and downstream proinflammatory gene transcription (28). Our results suggest that *nfkbia* expression in trained leukocytes is sustained and amplified upon secondary stimulation. Together with the reduced expression of *il1b*, *il6*, and *il10* in trained cells under LPS stimulation, this suggests a controlled inflammatory state that may limit cytokine responses while maintaining responsiveness to new stimuli through upregulation of CLRs.

In addition, *junb* expression in salmon leukocytes remained stable post-training but showed some increase after secondary stimulation. As part of the JUN family and the AP-1 complex, *junb* regulates cytokine expression in response to immune stimuli. In adipocytes of Atlantic salmon, LPS stimulation upregulates *nfkbia* and *junb*, indicating coordinated NF-κB inhibition and AP-1 activation during inflammation (41). In murine macrophages, it regulates *il1b* and other inflammatory genes following PAMP exposure and modulates macrophage activation (42). These findings support our observation that *junb* upregulation may contribute to sustained immune gene expression in stimulated leukocytes.

Cathelicidins are multifunctional antimicrobial peptides that play key roles in pathogen defence and immune modulation in teleosts. They function as both effector molecules with broad-spectrum antimicrobial activity and immunoregulatory mediators that influence inflammation and leukocyte activation (43). We did not observe a clear pattern in how training and re-stimulation affected the expression of *camp*, which contrasts with earlier reports of antimicrobial peptides induction by bacterial or endotoxin exposure in teleosts (27, 44). This suggests that cathelicidins play a limited role in the current study setup.

### 4.2 The expression of *pkm* and *sdha* in trained leukocytes suggests metabolic regulation after training and secondary stimulation

Trained immunity has been associated with metabolic adaptations in innate immune cells (4, 11, 45). However, mechanistic insights into immunometabolic regulation remain limited in teleosts compared to mammals. Accordingly, mammalian systems are used as a comparative framework for interpreting metabolic gene expression patterns observed in this study.

*Gglut1*, which encodes glucose transporter 1 (GLUT1) for glucose uptake, was significantly downregulated in trained salmon leukocytes 5 days post-exposure to β-glucan and showed comparable expression upon secondary stimulation. In mammals, GLUT1 facilitates glucose uptake in activated macrophages and neutrophils, as well as monocytes trained with oxidised low-density lipoprotein, supporting glycolysis and promoting proinflammatory activity (46–48). However, *glut1* downregulation has also been reported in murine macrophages when NF-κB signalling is inhibited (49), which aligns with the observed increase in *nfkbia* expression earlier in this study. As *glut1* expression did not change significantly upon secondary stimulation, its role in trained immunity in Atlantic salmon remains uncertain and warrants further investigation.

*Adpgk* encodes ADP-dependent glucokinase and initiates glycolysis under metabolic stress by using ADP instead of ATP. It has been identified as a key metabolic regulator gene in trained immunity in mammalian models (50). Although *adpgk* expression showed an increasing trend in trained cells, it was not significantly affected by primary training or secondary stimulation. This contrasts with earlier observation of *adpgk* upregulation in β-glucan-trained carp macrophages (12), suggesting that glucose processing may differ among species, and that training time and dosage could affect transcriptional responses.

*Pkm* encodes the enzyme Pyruvate Kinase M (PKM) and catalyses the final step in glycolysis, generating pyruvate and ATP. In this study, *pkm* expression increased in trained leukocytes after β-glucan training and remained elevated upon secondary stimulation, particularly in response to LPS. In human monocytes, oxidised low-density lipoprotein training upregulates both *PKM1* and *PKM2* gene expression upon secondary stimulation, suggesting that PKM has a critical role in regulating glycolysis in trained immunity (48). In mice, LPS stimulation similarly induces *pkm* expression and modulates cytokine profiles by suppressing IL-1β and increasing IL-10 production (51). These patterns parallel our findings, where LPS-stimulated trained leukocytes showed *pkm* upregulation alongside reduced *il1b* and stable *il10* expression. Our findings suggest that PKM regulation at the transcriptional level in salmon HK leukocytes may indicate engagement of glycolytic metabolism, with stronger activation under heterologous stimulation.

*Sdha* encodes the flavoprotein subunit A of succinate dehydrogenase (SDH) and was significantly upregulated in HK leukocytes at 5 days after β-glucan training. It remained elevated after secondary stimulation in trained cells, and this effect was significant under homologous conditions. SDH links the TCA cycle and oxidative phosphorylation by converting succinate to fumarate, thereby linking cellular respiration with metabolic intermediates (52). The role of SDH in trained immunity has been described in mammals but remains largely unstudied in teleosts. In β-glucan-trained human monocytes, SDH plays a central role in metabolic reprogramming, and its inhibition has been shown to modulate inflammatory cytokine production (52, 53). Our results suggest that metabolic regulation may involve both glycolysis and TCA cycle activity in trained salmon HK leukocytes, with *pkm* and *sdha* playing critical regulatory roles. These genes responded differently to the secondary stimulus but consistently showed higher expression in trained cells, indicating potentially stimulus-dependent metabolic modulation and transcriptional priming.

Although these results point to immune and metabolic modulation in β-glucan-trained leukocytes, they primarily reflect transcriptional regulation. A fixed resting period and single time-point evaluation may not fully capture the dynamic nature of leukocyte immune and metabolic responses. Moreover, because transcriptional changes do not reflect effector molecule production or activity, future time-series or dose–response studies combined with protein-level analyses would be required to resolve functional immune and metabolic outcomes.

### 4.3 Epigenetic markers are not significantly affected by β-glucan training

Epigenetic reprogramming is central to trained immunity and allows innate immune cells to enhance responses upon re-stimulation through changes in DNA, histone modification, and chromatin remodelling (4, 5, 11, 45).

*Dnmt1* encodes DNA methyltransferase 1 (DNMT1), which maintains DNA methylation during cell division and contributes to gene repression and pathogen-induced epigenetic regulation (54). In this study, *dnmt1* was significantly downregulated following β-glucan training, suggesting altered regulation of DNA methylation–associated pathways that may accompany immune activation. This finding contrasts with previous teleost studies, where *dnmt1* was upregulated in response to infection or stress, indicating stimulus-dependent regulation (54–56). Following secondary stimulation*, dnmt1* levels remained stable across treatments, although the downward trend with LPS stimulation may reflect altered epigenetic control in response to heterologous stimuli. The results suggest that modulation of *dnmt1* expression was detectable at 5 days post-exposure, but its functional implications following secondary stimulation remain unclear.

*Tet1* expression remained unchanged after β-glucan training but decreased in leukocytes upon secondary heterologous stimulation, although the patterns were inconclusive. In human macrophages, TET1 has been linked to immune-gene regulation by increasing hydroxymethylation of the *tnfa* promoter upon antigen exposure (57). However, it remains to be explored whether the low *tet1* expression levels under LPS stimulation relate to its regulation in response to familiar challenges rather than for heterologous responses in salmon HK leukocytes.

*Hdac3* expression remained unchanged after β-glucan training but showed an increasing trend in trained groups following secondary stimulation, with significant differences observed in response to LPS. HDAC3 is a histone deacetylase that is involved in histone modification and regulation of innate immune-gene expression in fish (54, 58). In common carp (*C. carpio*) and Nile tilapia (*Oreochromis niloticus*), β-glucan training is associated with marked upregulation of *hdac7* or *hdac11* (other histone deacetylase genes), particularly upon re-stimulation with the same stimulus, and has been interpreted as evidence of epigenetic involvement in trained immunity (15, 59). Our observations suggest that it may also play an important role in the response to secondary stimulation with heterologous stimuli or reflect stimulus-dependent regulatory responses rather than definitive epigenetic reprogramming in trained leukocytes.

*Ezh2* expression remained stable after both training and secondary stimulation in Atlantic salmon HK leukocytes, with no significant differences among treatments. EZH2 (enhancer of zeste homolog 2) is the catalytic subunit of the Polycomb Repressive Complex 2 (PRC2) complex and mediates H3K27 trimethylation to enforce long-term transcriptional silencing (54, 60). In mammalian macrophages, EZH2 suppresses pro-inflammatory genes like *il6* and *tnfa* through H3K27me3 deposition (60). This mechanism was absent in trained HK leukocytes stimulated with β-glucan and LPS, indicating that it may not be involved in the epigenetic-related transcriptional response of trained cells in the current study.

A key limitation of the present study is that it relies primarily on transcriptional profiling of selected epigenetic regulators and therefore cannot directly resolve specific DNA or histone modifications (e.g., H3K4me3, H3K27ac). While our findings indicate some sustained functional and transcriptional modulations consistent with trained immunity–like features, the underlying epigenetic and metabolic mechanisms remain to be elucidated. Future studies should incorporate targeted mechanistic approaches to address this gap. In particular, chromatin-based methods such as ATAC-seq or ChIP-seq could be employed to assess changes in chromatin accessibility and histone modifications at promoters and enhancers of immune-related genes. Integration of such chromatin-level analyses with transcriptome-wide approaches (e.g., RNA-sequencing), as applied in previous studies in fish (17, 18), would provide a more comprehensive framework to directly evaluate epigenetic reprogramming in trained Atlantic salmon leukocytes.

### 4.4 Cellular responses of trained HK leukocytes indicate increased phagocytosis accompanied by limited metabolic regulation after secondary stimulation

The most notable functional effect was increased phagocytosis in trained cells, particularly following LPS stimulation, suggesting enhanced antigen uptake in response to heterologous stimuli. Similar increases in phagocytic activity following β-glucan training have been reported in macrophages from common carp (*C. carpio*) and in neutrophils and macrophages from channel catfish (*I. punctatus*) (12, 18). Consistent with these findings, β-glucan priming has been shown to increase the phagocytic activity of head kidney macrophages in rainbow trout (*O. mykiss*) following both dietary and injectable administration, supporting phagocytosis as a phenotypic indicator of innate immune adaptation (61). In the present study, enhanced phagocytosis similarly reflects increased innate effector potential, especially upon LPS secondary challenge; however, its direct contribution to bacterial killing or pathogen clearance was not assessed.

Secondary stimulation with β-glucan induced ROS in both trained and untrained leukocytes, but there was no training-enhanced effect, suggesting direct stimulation. NO levels remained unchanged across treatments, indicating that β-glucan training did not influence NO production in this model. These results align with findings in macrophages from Nile tilapia (*O. niloticus*), where ROS levels remained stable after β-glucan exposure and re-stimulation, resulting in no signs of stress or overstimulation (59).

In contrast, other studies have reported elevated ROS and NO in trained cells. Trained turbot (*S. maximus*) neutrophils showed increased ROS and NO during bacterial infection (17), and haemocytes from crayfish (*Procambarus clarkii*) exhibited significantly higher ROS levels after pathogen exposure compared to untrained controls (62). Similarly, in common carp (*C. carpio*), trained macrophages showed heightened ROS production upon both homologous and heterologous re-stimulation, along with elevated basal ROS levels (12). These discrepancies likely reflect variations in cell type, training protocols, and species-specific responses, suggesting that elevated ROS and NO are not universal features of trained immunity, or that the magnitude of such a response is species- or cell-type-dependent and therefore plays a different role, as seen in trained leukocytes in Atlantic salmon.

Regarding metabolite production, studies in other fish models have reported increased extracellular lactate as a marker of glycolytic reprogramming (12, 17, 31, 59). However, our results showed no significant difference in intracellular lactate between trained and untrained leukocytes across treatments. In innate immune cells, glycolytic flux often increases upon activation and converts pyruvate from the TCA cycle into lactate (the Warburg effect) (46, 63). Lactate can serve as a metabolic fuel and a modulator of trained immunity (64). Most studies have identified elevated extracellular lactate as a hallmark of trained immunity. In our study, the absence of intracellular lactate accumulation may reflect rapid lactate export, diversion of metabolites toward mitochondrial pathways, or regulation of glycolytic flux by additional factors in trained salmon HK leukocytes.

As a key metabolite in trained immunity, fumarate contributes to metabolic and epigenetic reprogramming, and its glutaminolysis-driven accumulation is a hallmark of β-glucan-induced metabolic shifts and a potential driver of trained immune states (7, 12, 53). Lower fumarate levels under LPS stimulation in trained cells suggest reduced involvement during heterologous responses. Overall, our findings imply that trained salmon leukocytes may exhibit relative engagement of mitochondrial metabolism during homologous stimulation, while the role of fumarate appears limited or transient. This study did not assess other intermediates linked to trained immunity, such as succinate and itaconate, which have been well studied in mammal models. Furthermore, a single sampling time point in the current study may limit the capture of metabolite outputs. Thus, future studies should explore whether additional metabolic pathways and time-series investigation of metabolic products may contribute to trained immunity in fish (1, 3–5).

Beyond the limited changes observed in lactate and fumarate, a broader assessment of metabolic rewiring is warranted to fully capture the metabolic basis of trained immunity. These may include extracellular flux analyses (e.g., Seahorse assays) to measure real-time glycolytic rate and mitochondrial respiration, as well as targeted metabolomics to quantify intermediates across glycolysis, the TCA cycle, and related pathways. Stable isotope tracing (e.g., ^13C-glucose or ^13C-glutamine) would further enable tracking of metabolic flux and pathway utilisation, providing direct insight into how substrates are processed following β-glucan exposure. Together, these complementary approaches would provide a more comprehensive understanding of how β-glucan exposure influences metabolic programming and shapes trained immunity in Atlantic salmon leukocytes.

## 5 Conclusion

This study provides evidence that β-glucan exposure may induce trained immunity–like features in HK leukocytes of Atlantic salmon. We demonstrate that β-glucan exposure alters the transcriptional profile of HK leukocytes, with changes persisting for at least five days. These alterations result in distinct responses to both homologous and heterologous stimuli upon secondary stimulation.

Enhanced phagocytic activity emerged as the most prominent cellular response. In parallel, transcriptional analyses revealed early modulation of immune and metabolic pathways, as well as stimulus-dependent gene expression following secondary activation, together indicating a heightened functional state. Notably, significant differences between trained and untrained leukocytes were most evident five days post-training, suggesting sustained and stimulus-specific modulation of leukocyte responsiveness.

Although metabolic outputs such as lactate and fumarate showed limited variation, the differential expression of key immune- and metabolism-related genes indicates that β-glucan exposure influences selected pathways associated with innate immune training.

We acknowledge that several observed effects were moderate in magnitude and that the relatively small sample size (N = 5 fish) likely contributed to variability in some readouts. Increasing the number of fish and conducting *in vivo* experiments would help to better resolve these differences and further elucidate the underlying interactions. Nonetheless, the results suggest that β-glucan–related immune priming may operate in Atlantic salmon. Collectively, these findings support the hypothesis that trained immunity may occur in this species. While the precise underlying mechanisms remain to be fully elucidated, this study provides a strong foundation for future investigations.

## Data Availability

The raw data supporting the conclusions of this article will be made available by the authors, without undue reservation.
