# Supplementary material for: β-Glucan training induces stimulus-dependent immune and metabolic modulation in head kidney leukocytes that persists for several days in Atlantic salmon (Salmo salar L.)
Source: Front Immunol. 2026 May 7;17:1815436. doi: 10.3389/fimmu.2026.1815436 (PMC13189738; doi:10.3389/fimmu.2026.1815436)
Supplement: Supplementary file 1 [file Table1.docx]

Supplementary Material

**Supplementary Table 1. Gene selection for gene expression analysis by real-time quantitative PCR**

|  | **Gene symbol** | **Gene name** | **Primers sequence: forward and reverse**  **(5′-3′)** | **Function** | **Gene bank accession number** | **References** |
| --- | --- | --- | --- | --- | --- | --- |
| **Reference genes** | *18s* | 18S ribosomal RNA | F: GCCCTATCAACTTTCGATGGTAC R: TTTGGATGTGGTAGCCGTTTCTC | Ribosome function Protein synthesis | AJ427629 | [1] |
|  | *elf1a* | Elongation Factor 1-alpha AB | F: TGCCCCTCCAGGATGTCTAC R: CACGGCCCACAGGTACTG | Involved in translation | AF321836 | [3] |
|  | *ubi* | ubiquitin | F: AGCTGGCCCAGAAGTACAACTGTG R: CCACAAAAAGCACCAAGCCAAC | Protein ubiquitination Involved in translation | AB036060.1 | [3] |
| **Immune-related genes** | *il1b* | Interleukin 1 beta | F: CCCGTCCCCATTGAGACTAAA R: AGCAGGACAGGTAGAGGTTGGATC | Immune response and signaling | AY617117 | [2] |
|  | *il6* | Interleukin 6 | F: GGAGGAGTTTCAGAAGCCCG R: TGGTGGTGGAGCAAAGAGTCT | Immune response and signaling | XP_013998506.1 | [4] |
|  | *il10* | Interleukin 10 | F: GAACGCAGAACAACCACTTTAAATCT R: GATCTCTTTCTTCAGCTCGTGGAA | Immune response and signaling | EF165028.1 | [2] |
|  | *tnfa* | Tumor Necrosis Factor Alpha | F: CGTGCAGTGGAGAAAGGATGA R: AAGAAGAGCCCAGTGTGTGGG | Immune response  Mediating inflammation | [NM_001123590.1](http://www.ncbi.nlm.nih.gov/entrez/query.fcgi?cmd=search&db=nucleotide&doptcmdl=genbank&term=NM_001123590.1) | [2] |
|  | *clra* | C-type lectin receptor A | F: GACAACACAACACTGACAAACAAG R: GTGATCCTCCTGACTGATGATT | B-glucan receptor | AY572832.1 | [3] |
|  | *nfkbia* | Nuclear Factor kappa-light-chain-enhancer of activated B cells inhibitor (alpha) | F: CCATCCAAAGGGAACTGTATGAG R: ACATGTTACCTCTTCCTCATCAG | Inhibition of Nf-kB signaling  pathway, related to transcriptional signal | BT058522.1 | Designed |
|  | *junb* | Jun B proto-oncogene, AP-1 transcription factor subunit | F: TACTGCACTGTTGGGACAGC R: GTTCAGTATGCCCCGAGTGT | Enables transcription  factor binding Involved in the regulation of  cell population proliferation | CA056715.1 | [9] |
|  | *camp* | Cathelicidin | F: TGGAGGCTAGCAACAACCTGA R: GCTTGGCTTCTTCTTGTCCGA | Bacterial response, AMP | AY728057.1 | [2] |
| **Metabolism-related genes** | glut-1 | Glucose transporter 1 | F: CGCCAGCCCATCTTCATC R: GAAAACAGCGTTGATGCCAGA | Facilitated glucose transportation | AF247728 | [5] |
|  | *adpgk* | ADP-dependent glucokinase | F: GGTGCTCAGCTATATGTTGG R: CAGCATCTCGTGTAGTTTAGG | Involved in glucose metabolism Enables ADP-specific glucokinase activity | XM_014124004.2 | Designed |
|  | *pkm* | pyruvate kinase | F: TTATCAAGGGAAGTGGTACATCTG R: GATTGTCTTCATCACAGTTCTCC | Involved in the glycolytic process Enables pyruvate kinase activity | XM_014175388.2 | Designed |
|  | *sdha* | Succinate dehydrogenase complex, subunit A | F: AGAACCTTATGCTGAACGCT R: CCATACGGTCCTTGAAGTCCT | Involved in the tricarboxylic acid cycle | NM_001139616.1 | Designed |
| **Epigenetic-related genes** | *dnmt1* | DNA (cytosine-5-)-methyltransferase 1 | F: TGTGTCTTAGAGCGGATCAAGG R: TAGTCCGACACCTCCATCTTCT | Maintenance DNA methylation | XM_014203933.2 | [6] |
|  | *tet1* | TET methylcytosine dioxygenase 1 | F: GAGCAAATCATAGAGAAGGAGGA R: CTCTCCATACCTGTTCTCCA | DNA demethylation | XM_045722519.1 | Designed |
|  | *hdac3* | Histone deacetylase 3 | F: TAGAGCTTCTGAAGTACCACC R: CTGGAAAGAAGTAGTTCCCGT | Histone modification | XM_045723318.1 | Designed |
|  | *ezh2* | Enhancer of zeste 2 | F: ACTCTTTAGGGTTCTCATCGG R: ACTCTGAACTCATATACCTGTCTG | Histone H3K27 methyltransferase activity Involved in chromatin remodeling | XM_045702051.1 | Designed |

**Coefficients of variation (CV) for each gene were assessed separately for the two time points:
at 5 days post-training, CVs were 18s = 22.43 %, elf1a = 6.91 %, and ubi = 7.10 %;
at 24 hours post-secondary stimulation, CVs were 18s = 23.59 %, elf1a = 10.28 %, and ubi = 8.86 %.**

**References**

[1] Škugor, S., Škugor, A., Todorčević, M., Torgersen, J., Ruyter, B., & Krasnov, A. (2010). Exposure to lipopolysaccharide induces immune genes in cultured preadipocytes of Atlantic salmon. Fish & Shellfish Immunology, 29(5), 817–824. https://doi.org/10.1016/j.fsi.2010.07.026

[2] Seppola, M., Mikkelsen, H., Johansen, A., Steiro, K., Myrnes, B., & Nilsen, I. W. (2015). Ultrapure LPS induces inflammatory and antibacterial responses attenuated in vitro by exogenous sera in Atlantic cod and Atlantic salmon. Fish & Shellfish Immunology, 44(1), 66–78. https://doi.org/10.1016/j.fsi.2015.01.018

[3] Kiron, V., Kulkarni, A., Dahle, D., Vasanth, G., Lokesh, J., & Elvebo, O. (2016). Recognition of purified beta 1,3/1,6 glucan and molecular signalling in the intestine of Atlantic salmon. Developmental & Comparative Immunology, 56, 57–66. https://doi.org/10.1016/j.dci.2015.11.007

[4] Romero, A., Manríquez, R., Alvarez, C., Gajardo, C., Vásquez, J., Kausel, G., Monrás, M., Olavarría, V. H., Yáñez, A., Enríquez, R., & Figueroa, J. (2012). Prolactin-releasing peptide is a potent mediator of the innate immune response in leukocytes from Salmo salar. Veterinary Immunology and Immunopathology, 147(3–4), 170–179. https://doi.org/10.1016/j.vetimm.2012.04.014

[5] Ulvestad, J. S., Kumari, J., Seternes, T., Chi, H., & Dalmo, R. A. (2018). Studies on the effects of LPS, β-glucan and metabolic inhibitors on the respiratory burst and gene expression in Atlantic salmon macrophages. Journal of Fish Diseases, 41, 1117–1127. https://doi.org/10.1111/jfd.12806

[6] Beemelmanns, A., Zanuzzo, F. S., Xue, X., et al. (2020). The transcriptomic responses of Atlantic salmon (*Salmo salar*) to high temperature stress alone, and in combination with moderate hypoxia [Preprint]. Research Square. https://doi.org/10.21203/rs.3.rs-38228/v1

[7] Olsvik, P. A., & Søfteland, L. (2018). Metabolic effects of p,p′-DDE on Atlantic salmon hepatocytes. Journal of Applied Toxicology, 38, 489–503. https://doi.org/10.1002/jat.3556

[8] Emam, M., Eslamloo, K., Caballero-Solares, A., Lorenz, E. K., Xue, X., Umasuthan, N., Gnanagobal, H., Santander, J., Taylor, R. G., Balder, R., Parrish, C. C., & Rise, M. L. (2022). Nutritional immunomodulation of Atlantic salmon response to Renibacterium salmoninarum bacterin. Frontiers in Molecular Biosciences, 9, 931548. https://doi.org/10.3389/fmolb.2022.931548

[9] Martin, S. A., Douglas, A., Houlihan, D. F., et al. (2010). Starvation alters the liver transcriptome of the innate immune response in Atlantic salmon (*Salmo salar*). BMC Genomics, 11, 418. https://doi.org/10.1186/1471-2164-11-418
